# Changes in the dynamic characteristics of G-protein can alter the immune-protection efficacy of rabies virus vaccine

**DOI:** 10.1128/jvi.01954-24

**Published:** 2025-02-21

**Authors:** Chang-xu Chen, Xi Wang, Wen Su, Yuan Tian, Yu Gao, Dong-lan Liu, Hong Xiang, Bo-chuan Liu, Jin-li Shi, Yang Zhang, Dong Shen, Wen-zhi He, Li Yang, Chao Hong, Fan Wu, Lei-tai Shi, Yi-na Cun, Jian Zhou

**Affiliations:** 1 Bioproduct R&D Process Research Platform, Institute of Medical Biology, Chinese Academy of Medical Sciences & Peking Union Medical College 165063 , Kunming, China; 2 Biological Product Batch Issuance Laboratory, Medical Products Administration of Yunnan Province, Kunming, China; 3 National Institutes for Food and Drug Control 12540 , Beijing, China; University of North Carolina at Chapel Hill, Chapel Hill, North Carolina, USA

**Keywords:** rabies, vaccines, G-protein structure, molecular dynamics, immunogenicity

## Abstract

The efficacy of the G-protein is influenced by N-linked glycosylation, which serves as the sole immunogen of the rabies virus vaccine. However, achieving satisfactory immune-protection efficacy remains challenging, owing to the heterogeneous glycosylation of G-proteins. Within molecular dynamics, examining the impact of N-glycan heterogeneity on the structural characteristics of G-proteins provides insights into the relationship between antigens and the efficacy of rabies virus vaccines. Glycosylation is regulated by host cells. In rabies virus cultured in Vero cells (VRV), all N-glycosylation sites of the G-protein underwent modification. In contrast, rabies virus G-protein cultured in KMB17 cells (human diploid cell vaccine [HDCV]) was only modified by N-glycans at amino acid positions 247 and 319. Furthermore, treatment of VRV with de-glycosylation significantly improved its immune-protective efficacy, whereas de-glycosylation did not alter the immune-protective efficacy of HDCV. To support the impact of glycosylation on VRV efficacy, the structures and dynamics of G-proteins were analyzed using GROMACS. Specifically, the hydrophobicity, flexibility, and radius of gyration of the G-protein trimer in VRV were significantly altered by excessive hydrogen bonds formed by the three-branched hybrid glycan at the aa 319 site. These changes increase the instability of the G-protein trimer and may lead to a decrease in vaccine protective efficacy. Ultimately, we determined that N-glycan heterogeneity affects the immune-protection effect of antigen proteins by altering their dynamic characteristics, enhancing our understanding of the correlation between antigen structural characteristics and efficacy.

**IMPORTANCE:**

N-glycosylation of rabies virus glycoprotein dynamically regulates protein folding, stability, and antigenicity. Therefore, regulation of N-glycan modification is key to improving vaccine stability and protective efficacy. How the type and modification sites of N-glycans affect the protective efficacy of rabies vaccines remains unclear. Our research indicates that there are differences in the protective efficacy of rabies virus G-proteins modified with different N-glycans. Moreover, the modification of the three-branched hybrid glycan at the aa 319 site of G-protein significantly altered the hydrophobicity, flexibility, and radius, and increased its trimeric antigen instability through molecular dynamics demonstrations. These findings update the current understanding of the impact of glycans on vaccine antigenicity and develop a system to evaluate the stability of antigen glycoproteins based on molecular dynamics.

## INTRODUCTION

Rabies virus (RABV) is estimated to cause 59,000 human deaths annually in Africa and Asia as a fatal zoonotic encephalitis (1, 2). Several vaccines have been developed for use in humans to prevent mortality and morbidity when used as pre-exposure prophylaxis in high-risk individuals or post-exposure prophylaxis (3). Purified Vero cell vaccine (VRV) and human diploid cell vaccine (HDCV) have been widely used and studied. Currently, a large amount of experimental data supports the effectiveness of VRV and HDCV. However, HDCV is associated with a lower risk of local pain and fever (Sanofi Pasteur, France) (4, 5). Importantly, current research on rabies vaccines is still limited by sample size, diversity of measurable variables, and experimental methods, which may reduce the reliability of experimental results, especially when we use enzyme-linked immunosorbent assay (ELISA) to measure neutralizing antibody levels; antibody specificity can lead to heterogeneity of experimental results (5). There is still a lack of objective and effective methods to carefully evaluate differences in vaccine protective activity.

The glycoprotein is the only viral antigen with protective activity that is located on the surface of the rabies virus, and it also mediates receptor binding and membrane fusion during host-cell entry as a trimeric class III viral fusion protein (6–8). The ectodomain of glycoprotein contains 439 amino acids, which are the authentic binding region of receptors and form homotrimers called “spikes” (9). The G-ectodomain can be divided into three domains: the fusion domain (FD), pleckstrin homology domain (PHD), and central domain (CD) (9, 10). Crystal structure analysis revealed that changes in pH result in domain reorientations and reconstructions (11). Hence, studies on glycoproteins have described four conformational states: pre-fusion, early intermediate, late intermediate, and post-fusion (9, 12). The conformational changes of glycoproteins are believed to be related to immunogenicity, such as the pre-fusion form being the ideal immunogen, as its surface exposes major neutralizing antibody epitopes (11). In addition, the antigenic structure interacting with specific antibodies of the RABV G-protein (RABV-G) has been determined, including four major antigenic sites (I, II, III, and IV) (9). Antigenic site I is a highly conserved linear epitope that includes 226–231 amino acids (13). Antigenic site II contains two discontinuous regions (aa 34–42 and aa 198–200) linked through a disulfide bond (14). Antigenic site III includes aa 330–338, of which aa 330, 333, 336, and 338 are critical residues (15). Antigenic site IV is a linear epitope located at aa 261–264 (16). Within the *Lyssavirus* genus, viruses have been classified according to both genetic and antigenic data into phylogroups (17). And current vaccines based on inactivated preparations of classical RABV strains are highly effective against RABV of phylogroups, while the degree of cross-neutralization of RABV in other system groups is low (18, 19). However, Evans et al. showed that antigenic site II has immunodominance over other antigenic sites among different virus groups (20). This indicates that targeting more immunodominant and possible cross-neutralizing epitopes is essential for the development of vaccines that widely cover all RABV strains and *Lyssavirus* types.

The N-glycosylation of RABV-G is crucial not only for its correct conformation and function, but also for affecting the antigenic epitope. Most rabies viruses have two conserved N-linked glycosylation sequons at Asn37 and Asn319 in their glycoproteins (21). The G-protein can also have multiple glycosylation sites depending on the viral strain, such as Asn247 (22, 23). Previous studies have shown that N-glycosylation of Asn319 is important for the formation of a low pH-dependent fusion conformation and for virus particle production (24). In contrast, glycosylation of Asn247 results in reduced pathogenicity in adult mice (25). Furthermore, as a conserved glycosylation site, Asn37 is normally not effectively glycosylated, although glycosylation contributes to viral production (21, 24). On the other hand, glycosylation is not limited to maintaining the stability of a protein’s natural structure to exert its function (26, 27). Limiting the exposure of antigenic epitopes inhibits the ability of the immune system to recognize viral spikes (27, 28). The importance of N-glycosylation of envelope proteins with respect to immune evasion has been observed across many viruses, including hepatitis C, hepatitis B, Hendra, Newcastle disease, and herpes simplex virus (27). Therefore, many viruses prevent B cell- and T cell-mediated neutralization reactions by encoding N-linked glycan sites in genes under selective pressure from the host humoral immune system (29). The impact of glycosylation, both contributing to the exposure of antigenic epitopes and impeding antigen peptide presentation, underscores its role in adaptive immune activation. Although the stability and function of proteins regulated by glycosylation are widely known, little is known about how the protein structure is affected by glycans.

In recent years, methods for improving antigen stability based on molecular structures have played an important role in vaccine development (30). Glycan complexity has always been a challenge in protein structure and function analyses, mainly because of the different types of glycans that are stereoscopically linked within each glycosylation site (31). Currently, molecular dynamics (MD) simulations can reveal some characteristics of the influence of glycans on glycoprotein structure and elucidate protein stability in a given conformation (32, 33). For example, AMBER (34), GLYCAM (35), and CHARMM (36) are now used for the molecular dynamics analysis of glycoproteins. Such simulations provide key information regarding the effects of glycans on protein conformation. Given that glycoproteins not only play an important role in the success of rabies virus infection but are also the only antigenic targets, understanding the structure and effects of adding polysaccharides to this protein can provide important insights into the mechanism of extensive neutralizing antibody induction and highlight potential vaccine research directions. In this study, we constructed glycan profiles of CTN-1 rabies virus produced by Vero and KMB17 cells using mass spectrometry. We used molecular dynamics calculation tools to simulate the movement trajectory of the structure of the rabies virus before and after glycosylation, determined the changes in structural characteristics of glycoproteins, and linked their structural characteristics with immune-protective efficacy, which can achieve a more effective and computationally driven antigen design.

## RESULTS

### The CTN-1 of RABV was both glycosylated after being cultured in Vero cells and KMB17 cells

**Fig 1:**
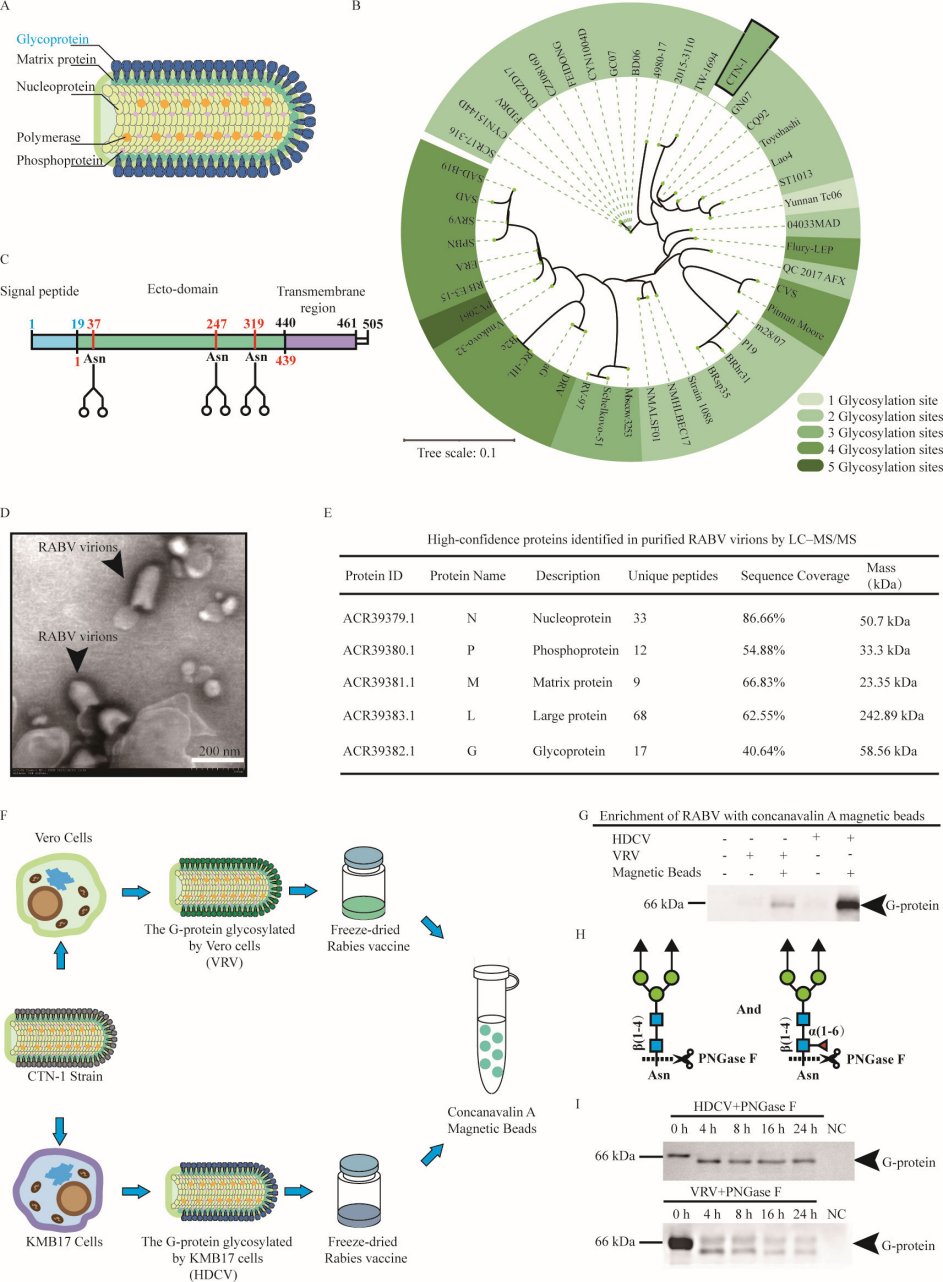
Identification and glycation detection of CTN-1 rabies virus. (**A**) The structure of RABV. (**B**) Comparison and evolution analysis of rabies subspecies gene families. (**C**) Glycosylation sites of rabies virus G-protein. (**D**) The image of CTN-1 strain of rabies virus by transmission electron microscopy (bar = 200 nm). (**E**) Coverage of protein sequence of CTN-1 strain of rabies virus. (**F**) The enrichment process of G-protein in freeze-dried rabies virus vaccine. (**G**) Specific detection of rabies virus G-protein. (**H**) Schematic diagram of PNGase F enzyme cleavage sites. (**I**) Molecular weight detection of G-protein after de-glycosylation.

The CTN-1 strain of rabies virus includes five proteins: nucleoprotein, phosphoprotein, matrix protein, large protein, and glycoprotein. Glycoprotein is the major antigenic protein that initiates the host’s adaptive immune response (Fig. 1A). The G-protein of RABV is a typical N-glycan-modified glycoprotein, and its glycosylation modification sites change with the evolution of the virus; the CTN-1 strain has three glycosylation modification sites as a laboratory-fixed strain used for preparing rabies virus vaccines (Fig. 1B). These N-glycosylation modification sites were located at amino acids 37, 247, and 319 of the ectodomain (Fig. 1C).

We prepared a CTN-1 rabies virus vaccine (HDCV) using human diploid cells (KMB17) for comparison with the commercial rabies virus vaccine of the CTN-1 strain proliferated in Vero cells (VRV). First, KMB17 produced rabies virions, as confirmed by transmission electron microscopy (TEM) (Fig. 1D). Meanwhile, the analysis of HDCV after trypsin digestion by liquid chromatography-mass spectrometry (LC-MS) showed that HDCV and the original protein sequence had high sequence coverage. The nucleoprotein, phosphoprotein, matrix protein, large protein, and glycoprotein sequence coverage was 86.66%, 54.88%, 66.83%, 62.55%, and 40.64%, respectively (Fig. 1E). The above results support that the HDCV vaccines prepared in this study were consistent with VRV.

It is difficult to directly use freeze-dried vaccines for the detection of G-protein glycosylation efficiency, as the use of human serum albumin can interfere with the G-protein signal (37). Thus, concanavalin A magnetic beads were used to enrich the RABV (Fig. 1F). G-proteins can be more easily bound with specific antibodies after enrichment by concanavalin A magnetic beads (Fig. 1G). PNGase F was used to verify whether the different cell cultures had glycosylation in the same virus strain. PNGase F cleaves the innermost GlcNAc and asparagine residues of high mannose, such as amidase, hybrid, and complex oligosaccharides from N-linked glycoproteins (Fig. 1H). By utilizing PNGase F to de-glycosylate glycoprotein, the molecular weight of the G-protein of VRV and HDCV decreased (Fig. 1H). Therefore, CTN-1 was glycosylated and cultured in Vero and KMB17 cell lines.

### Glycopatterns of CTN-1 virus were different when proliferated by Vero cells or by KMB17 cells

**Fig 2:**
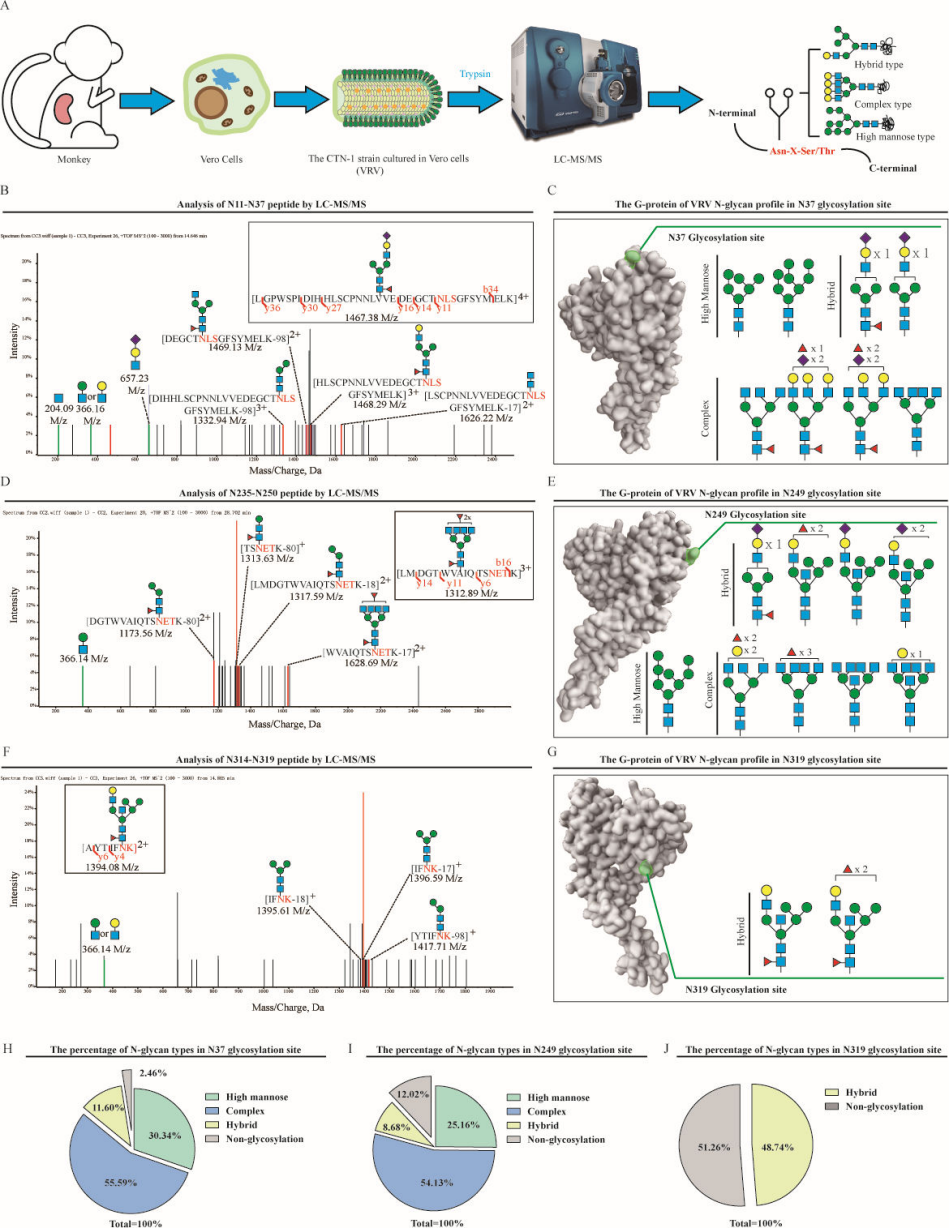
The N-glycan profile analysis on the G-protein of VRV. (**A**) Analysis process of G-protein and N-glycan. (**B, D, F**) Analysis of each glycosylation site by LC-MS/MS. (**C, E, G**) Glycan composition of each glycosylation site of the G-protein in VRV. (**H, I, J**) The proportion of various types of N-glycans at each glycosylation site in the G-protein of VRV.

Virions of CTN-1 that proliferated from the Vero cell system were extracted and characterized by LC-MS/MS after digestion with trypsin (Fig. 2A). The m/z data were analyzed and annotated using the GlycoMod online server. N-glycopeptides were screened by observing whether the characteristic fragment oxonium ion of the glycan appeared in the MS2 spectrum. Figure 2B, D, and F show the MS2 spectrum of glycopeptides, including [LGPWSPIDIHHLSCPNNLVVEDEGCTNLSGFSYMELK]^4+^, [LMDGTWVAIQTSNETK]^3+^, and [AYTIFNK]^2+^, in which the low-molecular-mass end was the signal of oxonium ions, such as m/z 204.09 [HexNAc + H]^+^ and m/z 366.14 [HexNAc + Hex + H]^+^. Information about the glycan as well as the cleavage of N-glycopeptides is shown in Fig. 2B, D, and F, b- and y-type cleavage provided detailed sequence and branching information, despite being dehydrated and dehydroxylated. Thus, the N-glycan composition was identified from oxonium ions and N-glycopeptide fragments. By considering the results from the LC-MS/MS, eight glycans, nine glycans, and two glycans from the Vero cell systems were identified at aa 37, aa 247, and aa 319, respectively (Fig. 2C, E, and G). After classifying the detected N-glycans and analysis by area normalization method, the complex type was mainly N-glycan at aa 37, with a ratio of 55.59%; the high-mannose type accounted for 30.34%, and the hybrid type only had 11.6%. In addition, 2.46% of G-protein was not glycosylated (Fig. 2H). Similar to aa 37, it also contained 54.13% complex glycan, 25.16% high mannose, and 8.68% hybrid glycan at aa 249. However, the non-glycosylation rate increased to 12.02% at aa 249 (Fig. 2I). Notably, only hybrid glycans were found at aa 319, with a glycosylation modification rate of 48.74% (Fig. 2J). In conclusion, all glycosylation sites of the G-protein of CTN-1 were modified by culturing in Vero cells. Among them, aa 37 and aa 249 are modified by different glycan types, whereas only aa 319 is modified by a hybrid glycan with low glycosylation efficiency.

**Fig 3:**
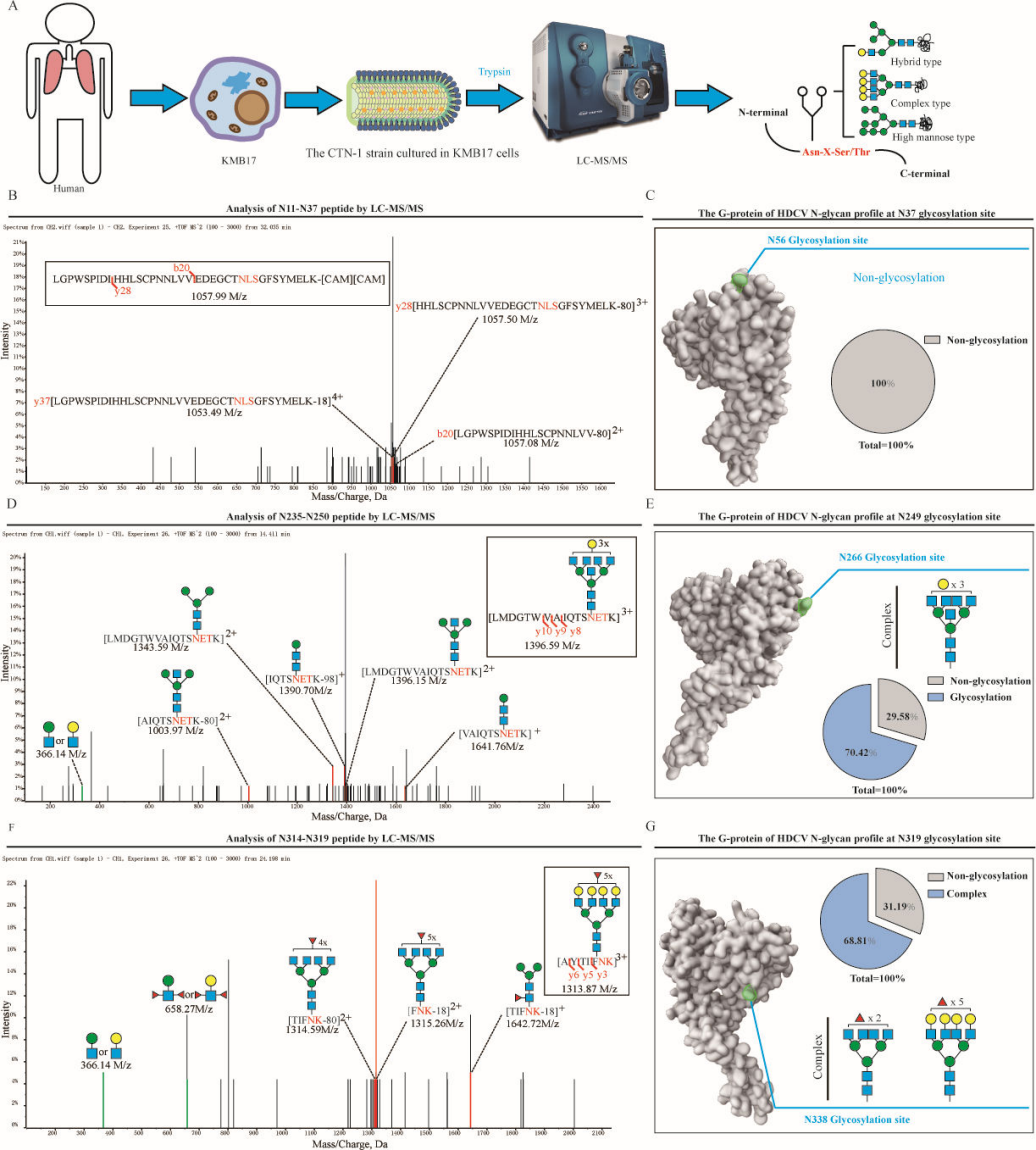
The N-glycan profile analysis on the G-protein of HDCV. (**A**) Analysis process of G-protein and N-glycan. (**B, D, F**) Analysis of each glycosylation site by LC-MS/MS. (**C, E, G**) Glycan composition and the proportion of various types of N-glycans at each glycosylation site in the G-protein of HDCV.

Similar to the process of analyzing the composition of N-glycan of VRV, virions of CTN-1 that were cultured in the KMB17 cell systems were characterized by LC-MS/MS after digestion with trypsin (Fig. 3A). However, we only found N-glycans at aa 249 and 319, both of which are complex type. At aa 37, only non-glycosylated peptides were confirmed, whereas only one five-branched complex glycan was found at aa 249, and two four-branched complex glycans were found at aa 319 (Fig. 3B, D, and F). The glycosylation efficiencies were 0%, 70.42%, and 68.81%, respectively (Fig. 3C, E, and G). Interestingly, the glycosylation pattern of rabies virus cultured in different cells showed significant differences, with the main difference being that the G-protein of VRV at aa 37 has glycosylation modification, while HDCV does not; the G-protein of VRV at aa 319 is modified by three-branched hybrid glycans, while HDCV is modified by four-branched complex glycans; the G-protein of VRV contains a variety of N-glycan types, while HDCV only has complex type modification.

### The efficacy of VRV vaccine was enhanced after de-glycosylation

**Fig 4:**
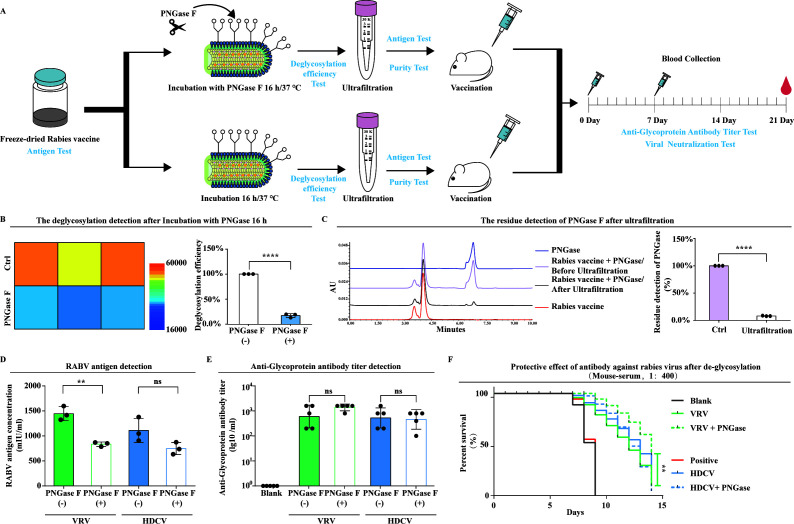
Immunogenicity testing of RABV vaccine before and after de-glycosylation. (**A**) Immunogenicity testing process. (**B**) The detection of de-glycosylation efficiency. (**C**) Purity detection of processed vaccines. (**D**) Determination of antigen in processed vaccines. (**E**) Detection of specific neutralizing antibody levels in mouse serum. (**F**) Detection of protective efficacy of specific neutralizing antibodies in mouse serum (1:400). (Data are presented as mean ± SD, *n* ≥ 3. *t*-test and log-rank test were performed for comparisons; ns, no significant difference, **P < 0.01, and ****P < 0.0001. The blank group refers to the treatment group that used mouse serum, which had not been immunized with rabies virus vaccine to neutralize CVS-11, whereas the positive control refers to the control group that directly injected CVS-11 into the mouse brain without mouse serum in panel F.

After completion of glycopattern analysis of the CTN-1 virus, the effect of glycosylation modification on vaccine immune-protective efficacy will be discussed below. Two different freeze-dried rabies virus vaccines (VRV, 4.945 IU/mL and HDCV, 4.944 IU/mL) were evenly divided into two parts, one of which was incubated with PNGase F at 37°C for 16 h. After removing excess glycosidases through ultrafiltration, antigen detection was performed, and finally used for mouse immunity; the other sample was incubated at 37°C for 16 h without the addition of PNGase F, and the mice were immunized after ultrafiltration and antigen testing. The mice underwent two immunization programs, and blood was collected on day 21 for antibody testing and virus neutralization experiments (Fig. 4A). To ensure the credibility of the process, we designed a detection method based on the ELISA framework, which uses specific antibodies to immobilize rabies virus particles and then combines fluorescent-labeled concanavalin A with the immobilized rabies virus to demonstrate its glycosylation removal efficiency. We compared the de-glycosylation efficiency of the same batch of vaccines, and the results of three independent repeated experiments showed that the freeze-dried rabies virus vaccine could stably remove over 83% of N-glycans after being re-dissolved according to the manufacturer’s instructions and incubated with PNGase F at 37°C for 16 h (Fig. 4B). Meanwhile, we purified the sample after enzyme treatment through ultrafiltration, which can remove more than 90% of the glycosidase residues detected by high-performance liquid chromatography (HPLC) (Fig. 4C). All processed samples were tested for antigen, and the results showed that only the VRV vaccine had a significant decrease after treatment, while the HDCV vaccine did not (Fig. 4D). Importantly, the binding antibody concentration in mouse serum did not significantly change after de-glycosylation of VRV and HDCV vaccines by antibodies titer detection (Fig. 4E). Furthermore, we diluted the CVS-11 strain by 2,000 times [median lethal dose (LD_50_) = 7.5] and conducted gradient neutralization experiments with the obtained mouse serum (1:200, 1:400, 1:800, 1:1,600, 1:3,200, 1:6,400). The results showed that when the dilution ratio of mouse serum was less than 400, the mice serum neutralization efficiency induced by de-glycosylation VRV was higher, and the potency of the VRV vaccine was improved (Fig. 4F).

### The molecular dynamics characteristics of G-protein trimer in VRV vaccine are more unstable because of N-glycosylation

**Fig 5:**
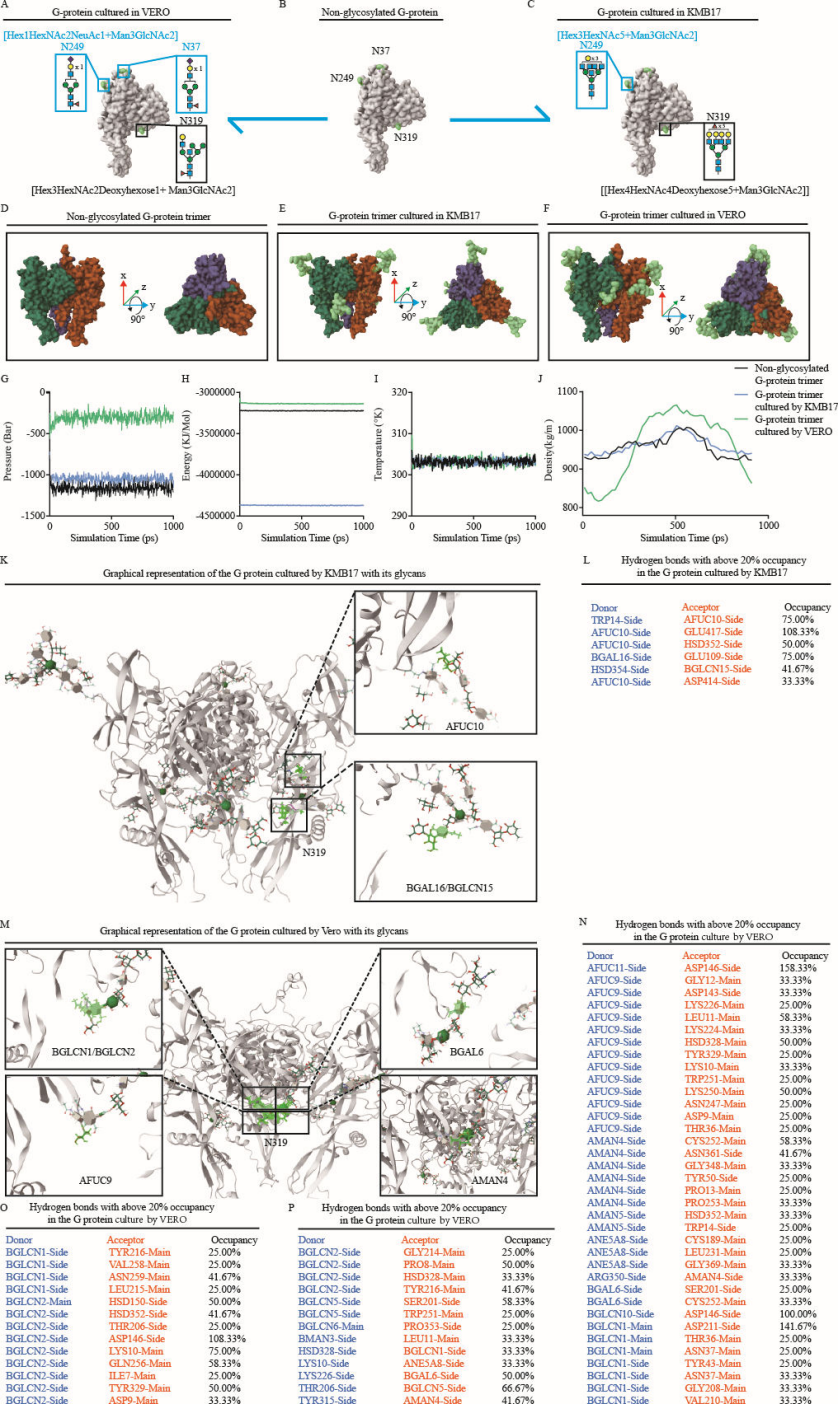
Hydrogen bonds with above 20% occupancy in the G-protein cultured with different cell lines. (**A**) N-glycan structure used for modeling G-protein in VRV. (**B**) G-protein modeling without N-glycan structure. (**C**) Glycan structure used for modeling G-protein in HDCV. (**D–F**) G-protein trimeric models under different cell culture systems. (**G–J**) The pressure, energy, temperature, and density during the MD. (**K**) Graphical representation of the G-protein cultured by KMB17 cells. (**L**) Hydrogen bonds with above 20% occupancy in the G-protein cultured in KMB17 cells. (**M**) Graphical representation of the G-protein cultured by Vero cells. (**N–P**) Hydrogen bonds with above 20% occupancy in the G-protein cultured in Vero cells. (The “HSD” in the data graph refers to the charged forms of histidine).

Considering that the CTN-1 strain carries different and complex glycan patterns that proliferate in different cells and exhibit different immune-protective effects after de-glycosylation, we used molecular dynamics to study the underlying molecular mechanisms. The Glycan Reader and Modeler of the CHARMM-GUI tool were used to model the RABV glycoprotein. Protein Data Bank (PDB) models of RABV G-protein (7U9G) without oligosaccharide chains were obtained from the Research Collaboratory for Structural Bioinformatics. Here, the oligosaccharide chains of [Hex_1_HexNAc_2_NeuAc_1_+Man_3_GlcNAc_2_], [Hex_1_HexNAc_2_NeuAc_1_ + Man_3_GlcNAc_2_], and [Hex_3_HexNAc_2_Deoxyhexose_1_ + Man_3_GlcNAc_2_] were added to amino acids 37, 249, and 319 to simulate the structure of the G-protein from VRV (Fig. 5A); the glycans of [Hex_3_HexNAc_5_ + Man_3_GlcNAc_2_] and [Hex_4_HexNAc_4_Deoxyhexose_5_ + Man_3_GlcNAc_2_] were added to amino acids 249 and 319 to simulate the G-protein in HDCV (Fig. 5C). G-protein without glycosylation modification was used as a blank control for reference (Fig. 5B).

The modeling process was as described by Coutinho et al. (32) The final model for the MD simulation was visualized using visual molecular dynamics (VMD) (Fig. 5D through F). To proceed with the MD simulation, the energy, temperature, pressure, and density of the RABV G-proteins were analyzed to perform this correction. The results showed that the energy, temperature, and pressure of different G-protein trimers were stable (Fig. 5G through I); only the density of the G-protein trimers in the VRV showed significant fluctuations compared to the others (Fig. 5J). The hydrogen bonds involving a glycan chain with a protein chain found in the simulation differed between VRV and HDCV, although the hydrogen bonds were formed by glycans located at aa 319. The G-protein of VRV can form 51 hydrogen bonds because of the hybrid glycan at aa 319, involving most of the ectodomain (Fig. 5M through P). In contrast, the complex glycan at aa 319 of the G-protein in HDCV only formed six hydrogen bonds (Fig. 5K and L).

**Fig 6:**
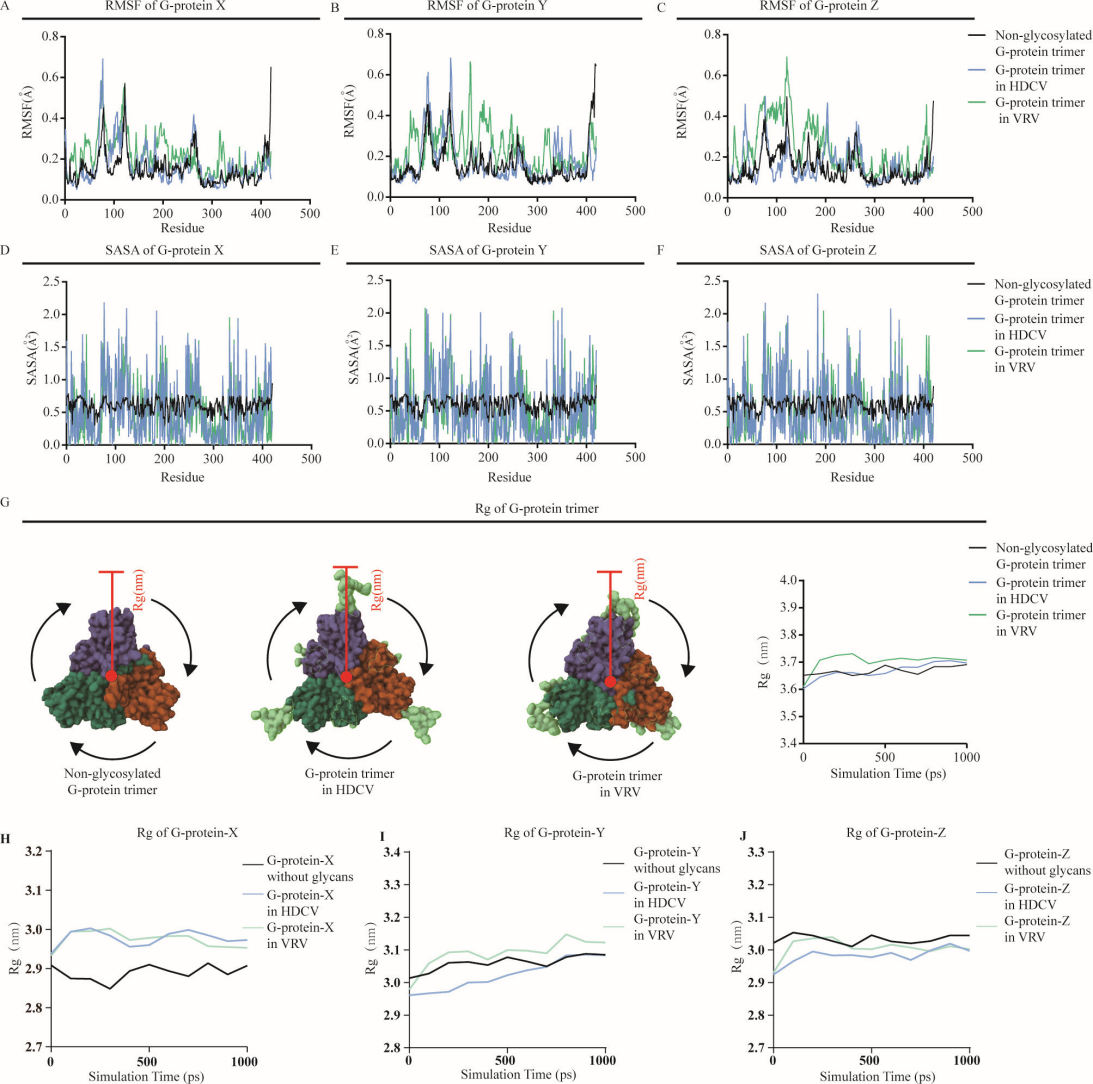
Molecular dynamics simulation of G-protein carrying N-glycans. (**A–C**) The root mean square fluctuation (RMSF) per residue of each subunit in G-protein trimer. (**D–F**) Representation of solvent accessibility for each amino acid residue of each subunit in G-protein trimer. (**G**) Comparison of gyration radius of G-protein trimers with different sugar chains. (**H**) Comparison of gyration radius of G-protein X between G-protein without glycans, G-protein in HDCV, and G-protein in VRV. (**I**) Comparison of gyration radius of G-protein Y between G-protein without glycans, G-protein in HDCV, and G-protein in VRV. (**J**) Comparison of gyration radius of G-protein Z between G-protein without glycans, G-protein in HDCV, and G-protein in VRV.

The molecular flexibility of the G-protein changed after glycosylation, as determined by calculating the root mean square fluctuation per residue, especially the G-protein in the VRV. We found that almost every subunit of the G-protein from VRV had higher molecular flexibility around aa 50, aa 200, and aa 300 in its sequence, and almost every subunit of the G-protein from HDCV had a molecular flexibility curve that was highly consistent with the non-glycosylated G-protein, although the G-protein Z subunit from HDCV exhibited high molecular flexibility near amino acids 50 and 200 (Fig. 6A through C). Moreover, the surface area of solvent exposure was significantly changed after glycosylation, which was reflected in the solvent-accessible surface area (SASA) measurements of G-protein cultured in both VRV and HDCV (Fig. 6D through F). During the gyration radius detection process, we found that the gyration radius of the G-protein trimer from VRV increased during MD simulation. In contrast, the radius of gyration curve for the G-protein trimer cultured in HDCV was closer to that of the glycosylated G-protein trimer (Fig. 6G). It is worth noting that when the dynamic characteristics of G-protein monomers are observed separately, even the radius of G-protein monomer Y in VRV was significantly higher than the other two groups of G-protein Y monomers. But the radius curve of VRV G-protein monomer Z was smaller than the control G-protein monomer. Therefore, glycosylation did not necessarily increase the radius of G-protein monomer; it may also decrease the radius of G-protein monomer (Fig. 6H through J). The radius of gyration was an important parameter for describing the density of protein structures; a smaller radius indicates a denser protein structure. Thus, the increase in the radius of G-protein trimer suggested the risk of loss of antigen structural stability.

## DISCUSSION

It is challenging to elucidate the impact of glycans on protein folding and function because of the dynamic properties and heterogeneity of glycan structures (38–40). The fact that dynamic changes in glycosylation type and occupancy rate can regulate protein folding suggests that not all glycosylation sites are necessary to achieve the correct natural conformation of proteins (41). Viruses constantly evolve to cope with host immune pressure, and achieving immune evasion to complete infection is their goal. Therefore, glycosylation provides an effective immune escape mechanism for viruses, and most neutralization reactions can be avoided by forming a glycan shield on the surface of membrane proteins (42, 43). However, not all glycosylation is successful, and most glycosylation modifications often have the opposite effect, such as compared to fully glycosylated hemagglutinin (HA), replacing a single N-acetylglucosamine to each N-glycosylation site of influenza virus HA can produce better cross protection (44). For inactivated viral vaccine products, glycosylation is required, which cannot mask antigen epitopes and is more prone to exposure, as it can help vaccines to be more protective. However, to date, there is a lack of research in this field. In this work, we first analyzed the sequence characteristics of the rabies virus G-protein and found the G-protein has three glycosylation sites, two of which are considered conservative sites (45). Conservative glycosylation sites will provide targets for the induction and occurrence of widespread neutralizing antibodies, which may be a direction for the development of effective vaccines. By comparing peptide sequence coverage and TEM detection, we confirmed that the cultured rabies virus was a CTN-1 strain. Because of the interference of human serum albumin components in freeze-dried rabies virus vaccines with the signal of G-protein, we enriched rabies virus from freeze-dried rabies virus vaccines using lectin magnetic beads and verified that the rabies virus was glycosylated in vaccines produced under different cell culture systems.

Wojczyk et al. characterized the recombinant G-proteoglycan profile of CHO cell lines. However, in their study, aa 37 could not be effectively glycosylated (46), which is similar to the rabies virus G-proteoglycan profile cultured in KMB17 cells, where the aa 37 position lacks glycosylation modification. Simultaneously, the rabies virus G-protein cultured in KMB17 cells exhibited a higher degree of glycan branching at positions 249 and 319, such as heterozygous glycans with four or five branches and fewer types of features. In contrast, the rabies virus G-protein cultured in Vero cells has a more complex polysaccharide profile, exhibiting not only glycosylation modifications at position 37 but also various N-glycan modifications at positions aa 37 and aa 249, including high mannan, complex, and hybrid glycan modifications. It is important to note that at position aa 319, two rabies viruses from different culture systems exhibited surprising single-type polysaccharide modification characteristics in their G-proteins. The rabies virus G-protein cultured in Vero cells contained only three-branched heterozygous glycans, while the rabies virus G-protein cultured in KMB17 cells contained only four-branched complex glycans. Previous studies have suggested that glycosylation between different sites will affect each other, although the mechanism behind this is not yet known (46). Notably, the position aa 319 was considered an important glycosylation site that affects G-protein folding (24, 46).

Glycosylation helps regulate the antigenic properties of glycoproteins, and may “inactivate” peptide epitope or may be required for its reactivity with the antibody, depending on the structure of the antigenic site and antibody fine specificity (47). Evidence suggests that glycosylated peptides can be bound and presented by major histocompatibility complex (MHC) class I or II molecules and trigger glycoside-specific T cell clones (48–50). To explore the effects of different glycan patterns on the efficacy of inactivated viruses, we attempted to analyze these effects using PNGase F to eliminate the glycan carried by the G-protein on the surface of inactivated rabies virus. However, because of the difficulty in accurately unifying antigen levels between vaccines and vaccine products, animal experiments are limited by individual differences and other factors, making it impossible to scientifically and directly compare efficacy differences between the two vaccine products. Therefore, we compared the titers of anti-G-protein antibodies before and after vaccine de-glycosylation in mouse serum through antibody titer detection. And the results showed that the de-glycosylation of VRV or HDCV did not significantly affect the production of anti-G-protein antibodies in mice. However, we found that VRV showed a significant increase in antibody neutralization efficacy after de-glycosylation when we tested the virus neutralization efficiency through mouse neutralization experiments, despite the titer of anti-G-protein antibodies not being significantly different before VRV de-glycosylation. The above results indicate that glycans limited the immune protection of rabies virus vaccines produced by Vero cells.

To understand how glycosylation limited the immune protection of rabies virus vaccines, we used MD to simulate the motion trajectory of G-protein trimer-carrying glycans and characterized their dynamic characteristics to better understand the mechanism by which glycans affect the protective effect of antigen proteins. After analysis, we found that when the sugar chain at aa 319 is a three-branched hybrid glycan, a large number of hydrogen bonds are formed inside the G-protein, whereas when the glycan is a four-branched complex type, there are only a few hydrogen bonds. While the main chain hydrogen bonding is important for protein folding, defining the secondary structural elements, hydrogen bonding between the side chains is crucial to protein-protein interactions (51). The formation of a large number of hydrogen bonds poses a challenge in maintaining the conformational stability of G-proteins. Therefore, when we delved deeper into the molecular flexibility and solvent contact area of G-protein, we found that G-protein cultured in Vero cells was more flexible than G-protein cultured in KMB17 cells, and the flexibility characteristics of G-protein cultured in KMB17 cells were closer to the original conformation, although both underwent drastic changes in their solvent contact surface area after carrying sugar chains. These changes may make it difficult for the G-protein to maintain its trimeric structure, thereby reducing the probability of effective acquisition of antigen structural features. Because neutralizing antibodies against rabies virus typically target the trimeric structure of G-protein (52), the G-proteins that cannot maintain their trimeric structure are unable to provide the host immune system with more information on neutralizing target sites, and the ability of antibodies to neutralize viruses may significantly decrease; thus, the immune protection efficacy of vaccines cannot be guaranteed.

Finally, the results of this study highlight the importance of understanding the effects of N-glycosylation on the structure and stability of antigenic proteins, with immediate consequences for vaccine production. Indeed, earlier work shows that compared with vaccination of the traditional influenza vaccine with complex glycosylation from eggs, the mono-glycosylated split-virus vaccine provided better cross-strain protection against a lethal dose of virus challenge in mice (44, 53). However, whether glycosylation simplification is beneficial for enhancing immunogenicity remains unclear. Based on the existing G-protein construction and simulation techniques, we can better understand the impact of different glycan profiles on the structure of antigen proteins, thereby linking them with the efficacy of vaccines. These antigen proteins can be designed to contain multiple or one type of glycan and can be expressed through cell engineering. Our results show that considering the effects of N-glycosylation on protein structural stability and dynamics in the context of specific protein sequences may be the key to understanding which glycan is more suitable for enhancing immune protection efficacy, which is important for the development of vaccine science.

## MATERIALS AND METHODS

### Vaccines and strain

The rabies virus freeze-dried vaccine produced by Vero cells was purchased from the China Center for Disease Control and Prevention, and the rabies virus freeze-dried vaccine produced by KMB17 cells was produced in the process research center of the Institute of Medical Biology, Chinese Academy of Medical Sciences. The above vaccine was produced by the CTN-1 strain. The CTN-1 was a fixed strain used in the production of rabies virus vaccines in China and sourced from the Institute of Medical Biology, Chinese Academy of Medical Sciences. The CTN-1 strain of rabies virus is a laboratory strain isolated, domesticated, attenuated, established, and applied to the brain tissue of a rabies patient. This strain meets the requirements and quality standards of national and WHO human vaccine production, and has achieved industrial production.

### Enrichment and purification of rabies virus

In this experiment, BeyoMag concanavalin A magnetic beads (P2156, Beyotime) were used to enrich and purify rabies virus particles and G-protein from freeze-dried rabies virus vaccine. The binding buffer, elution buffer, and wash buffer were prepared according to the instructions. First, the vaccines were dissolved in phosphate buffered saline (PBS). The magnetic beads were washed twice with an appropriate amount of binding buffer 10 times the volume. Then, the activated magnetic beads were added to the dissolved vaccine sample at a ratio of 10 µL to 250 µL, placed on a rotary mixer, and incubated at room temperature for 30 min. The sample was placed on the magnetic frame to separate for 1 min. The supernatant was removed, and the beads were washed three times with 0.5 mL of wash buffer. Finally, the 50 μL–250 μL elution buffer was added to each sample. After incubation at room temperature for 30 min, the supernant was collected and either used for Western blotting (WB) detection or placed in -80℃ storage.

### Protease digestion of trypsin

The ultrafiltration tube enzymolysis method was used for protein digestion; the sample solution (containing 100 µg virus protein) obtained in the previous step was added into the 10 kDa ultrafiltration spin columns (FUF051, Sartorius) and centrifuged at high speed until the filter membrane was dried. The 90 µL denaturant 7 M guanidine hydrochloride (5010-OP, Sigma) and 2 µL 1 M DTT (D9779, Sigma) were added to the columns and incubated at 45°C for 30 min. The 5 µL iodoacetamide (I6125, Sigma) was also added to the columns and incubated at room temperature in the dark for 30 min. Then, the solution in the columns was replaced with 50 mM Tris-HCl (pH 7.6, ST776, Beyotime) by centrifugation. Finally, the trypsin (1:50 mass ratio, P14209, Beyotime) was added in the columns, the volume was fixed to 100 μL with water, and the mixture was incubated at 37°C overnight. The next day, the sample was heated at 100°C for 10 min after treatment to terminate the reaction, and LC-MS/MS was used for detection.

### Glycopeptide and coverage analysis by LC-MS/MS

The SCIEX triple Quad 5500 LC-MS/MS system was used to analyze glycopeptide and coverage. The Waters Acquisition UPLC BEH C18 column (1.7 µm, 2.1 mm × 100 mm) was used for peptide separation. The specific method was as follows: mobile phase A, 98% water (Millipore Direct-Q 3 UV Water Purification System), 2% acetonitrile (1.00029, Sigma), and 0.1% trifluoroacetic acid (TX1276, Sigma); mobile phase B, 98% acetonitrile, 2% water, and 0.1% trifluoroacetic acid. The column temperature was 50°C, and the injection volume was 15 μL; separation gradient: 2% mobile phase B, 2 min; 35% mobile phase B, 36 min; 90% mobile phase B, 1 min; 90% mobile phase B, 4 min; 2% mobile phase B, 3 min; 0% mobile phase B, 4 min. The information dependent acquisition (IDA) and electron activated dissociation (EAD) fragmentation mode was adopted for analysis. The main time of flight mass spectrometery (TOF-MS) primary scanning and secondary scanning parameters were as follows: spray voltage, 5,500 V; TOF start mass, 200 m/z; TOF stop mass, 2,000 m/z; accumulation time, 0.1 s; source temperature, 450°C; declustering potential, 80 V; collision energy, 10 V; time bins to sum, 8; Q1 resolution, unit; electron beam current, 5,500 nA; Zeno trap, ON; Zeno trap threshold, 100,000 cps; start mass, 100 m/z; stop mass, 3,000 m/z; accumulation time, 0.1 s; electron KE, 1 eV; enhanced product ion dynamic fill time choosing (ETC), dynamic; time bins to sum, 8; EAD RF, 100 Da; EAD reaction time, 10 ms.

All MS/MS spectral data were searched using the SCIEX OS and analyzed by the online server Expasy. The Glycomod tool of Expasy was used to screen for glycopeptides that may contain glycan, and the screened glycopeptides were validated by secondary mass spectrometry using SCIEX peak view. The PeptideMass tool of Expasy was used to obtain the theoretical molecular weight of glycopeptide after trypsin digestion, and FindPept was used to identify peptides that result from specific cleavage of proteins from experimental masses.

### Electron microscopy of purified RABV particles

The rabies virions were examined by TEM to observe their purity and morphology with the negative staining method as described by Brenner with moderate adjustment (54). Virus particle was observed under H-7650 Hitachi transmission electron microscope at 80 kV.

### De-glycosylation and Western blot analysis

The purified virion preps were de-glycosylated to detect the glycosylation of G-protein. Aliquots of purified virus containing 100 µL G-protein were de-glycosylated with PNGase F (P2318S, Beyotime, China) as recommended by the manufacturer. For Western blot analysis, the proteins were transferred to a polyvinylidene fluoride (PVDF) membrane (FFP24, Beyotime, China) which was then probed with specific RABV glycoprotein polyclonal antibody (MAb0166, NanJing MiaoDi, China) and horseradish peroxidase (HRP)-labeled Goat Anti-Mouse IgG(H + L) (A0216, Beyotime, China). Protein bands were observed using an ChemiDoc Go imaging system (BIO-RAD, USA).

### HPLC method of purification efficiency after de-glycosylation

The Waters Alliance HPLC system with 2996 PDA detector was used to purity testing. The XBdridge BEH SEC column (200 Å, 3.5 µm, 7.8 mm × 150 mm) was used to separate the sample. The mobile phase was 100 mM sodium phosphate salt. The column temperature was room temperature, the injection volume was 20 µL, and the isocratic elution was 30 min with flow rate of 0.8 mL/min. The software Empower 3 was used for data analysis.

### Antigen, neutralizing antibody, and de-glycosylation efficiency detection

ELISA was used for detecting antigen, antibody levels, and de-glycosylation efficiency. The antigen detection process was as follows: the captured antibodies (mouse monoclonal antibody, MAb0322, NanJing MiaoDi, China) were added in a microplate (Corning) and incubated overnight. The supernatant was removed and wells were added with 3% bovine serum albumin (BSA; Sigma-Aldrich) in PBS for an additional hour at room temperature. Wells were washed three times with PBS and then incubated with 1:200 virus samples for 1 h at room temperature. Plates were washed three times with PBS. The HRP-labeled detection antibodies (1:5,000, Mouse monoclonal antibody, Mab0166, NanJing MiaoDi, China) were added into the microplate and incubated at room temperature for 30 min. Plates were washed three times with PBS, then developed with 3, 3′,5 ,5′-tetramethylbenzidine (TMB)-ELISA substrate solution, and quenched with 1 M sulfuric acid. Plates were read at 450 nm on an ELISA reader.

For anti-RABV antibody level detection, the captured antibodies (mouse monoclonal antibody, MAb0322, NanJing MiaoDi, China) were added into a microplate and incubated overnight. The supernatant was removed and wells were added with 3% BSA (Sigma-Aldrich) in PBS for an additional hour at room temperature. Wells were washed three times with PBS and then incubated with 1 g/mL recombinant G-protein (AK100H1, ABMax, China) for 1 h at room temperature. Plates were washed three times with PBS. The antiserum was added into the microplate and incubated at room temperature for 30 min. Plates were washed three times with PBS and then incubated with 1:5,000 goat anti-mouse HRP (goat polyclonal antibody, A0216, Beyotime, China) for 30 min at room temperature. Plates were washed three times with PBS, then developed with TMB-ELISA substrate solution, and quenched with 1 M sulfuric acid. Plates were read at 450 nm on an ELISA reader.

The microplate wells were incubated with 1 μg/ml capture antibodies (mouse monoclonal antibody, MAb0322, Nanjing Miaodi, China) overnight to detect the efficiency of de-glycosylation. The supernatant was removed and wells were added with 3% bovine serum albumin in PBS for an additional hour at room temperature. Then, wells were washed three times with PBS and then incubated with the sample with or without de-glycosylation for 1 h at room temperature. Plates were washed three times with PBS. The fluorescein-labeled lectins (1:500, Concanavalin A-Fluorescein, FL-1001-25, Vector Lab, USA) were added in wells for 30 min at room temperature. Plates were washed three times with PBS and read at 521 nm with 495 nm excitation light on a Perkin Elmer Ensight ELISA reader.

### Mouse neutralization test

Virus neutralization tests were performed according to the Chinese Pharmacopoeia. The 100 µL rabies virus vaccines with or without de-glycosylation (1 IU/mL) were injected into the right thigh muscle of mouse on day 0 and day 7, respectively. The mouse serum was collected on day 21 for antibody testing (ELISA) and neutralization experiments [mouse neutralization text (MNT)].

The standard virus (CVS-11, LD_50_ = 7.5) was diluted with PBS (1:2,000). The 0.5 mL mouse anti-serum (1:200, 1:400, 1:800, 1:1600, 1:3200, 1:6400) was added in 0.5 mL standard virus dilution and incubated in a 37°C water bath for 1 h after being mixed. Mice weighing 10 g–12 g were used in the experiment, with six mice in each treatment group. The mice were injected with 0.03 mL virus suspension, which was incubated with mouse antiserum. After a mouse was taken out, it was held in the left hand; the mouse’s head was pinched by the thumb and index finger, and its body was gently pressed with the left palm. Alcohol was used to disinfect the heads of the mice. A syringe was inserted into the temporal region of the mouse (slightly toward the ear at the midpoint of the line connecting the eye and ear root), entering the cranial cavity, with the needle inserted 2–3 mm. The incidence and mortality of the mouse were observed daily for 14 days. The mice that died within 4 days after inoculation were counted as non-specific deaths, and mice that died 5 days after inoculation were counted as infected.

### Animal anesthesia and euthanasia

Specific pathogen free (SPF)-grade mice weighing 11–20 g and aged 2–7 weeks were used in the experiment. Six mice were housed in a cage and provided with clean water and food to maintain health and energy daily. In this experiment, the experimental animals at the end of the experiment and the humane endpoint were anesthetized and euthanized. The experimental animals at the humane endpoint exhibited typical symptoms of rabies virus infection, such as drowsiness, weight loss, loss of appetite, and body tremors. In this experiment, the inhalation drug method was used for anesthesia, which can minimize the fear, anxiety, and pain of animals as much as possible. Then, the cervical dislocation method was used to ensure animal death. Using a mixture of 3%–5% isoflurane and oxygen, the animal was placed in an anesthesia box to rapidly lose consciousness. After the loss of muscle tone in the animal was confirmed, ventilation was continued for 2 min, and euthanasia was completed by cervical dislocation.

### Molecular dynamics simulation methods

The molecular dynamics method was established by Coutinho et al. (32). The models were based on PDB 7U9G. Mass spectrometric data were used to determine the most frequent glycan structures in each glycosite. For this work, CHARMM-GUI was used due to its web-based intuitive interface for the addition of glycans, an integrated tool for MD model preparation. After setting the periodic boundary conditions and selecting the “force field” CHARMM36m to be used for topology generation and inserting standard parameters for the equilibration of the system, the CHARMM-GUI will generate input files for several MD simulation programs. Also, the GROMACS software was used for our simulations. The .mdp output from CHARMM-GUI can be used as input for the simulation. The trajectory file was analyzed using the VMD v.1.9.4. to perform trajectory visualization and other analyses to evaluate the behavior and interactions of the different segments of the glycoproteins along the simulation.

### Data statistical analysis

Data were analyzed using GraphPad Prism (v.7, Prism), and statistical significance was determined using *t*-test for unpaired experiments (two-tailed). *P* < 0.05 was considered to indicate statistically significant differences between groups. The resulting data are presented as means ± SEM from at least three independent experiments.

## Data Availability

The data generated or analyzed during this study are available from the corresponding author upon reasonable request. The mass spectrometry proteomics data have been deposited to the ProteomeXchange Consortium (http://proteomecentral.proteomexchange.org) via the iProX partner repository with the data set identifier PXD059482.
